# High mortality due to sepsis in Native Hawaiians and African Americans: The Multiethnic Cohort

**DOI:** 10.1371/journal.pone.0178374

**Published:** 2017-05-30

**Authors:** Michelle L. Matter, Yurii B. Shvetsov, Chase Dugay, Christopher A. Haiman, Loic Le Marchand, Lynne R. Wilkens, Gertraud Maskarinec

**Affiliations:** 1 University of Hawaii Cancer Center, Cancer Biology Program, University of Hawaii, Honolulu, Hawaii, United States of America; 2 University of Hawaii Cancer Center, Epidemiology Program, University of Hawaii, Honolulu, Hawaii, United States of America; 3 Department of Preventive Medicine, Keck School of Medicine, and Norris Comprehensive Cancer Center, University of Southern California, Los Angeles, California, United States of America; University of Hawai'i at Manoa College of Tropical Agriculture and Human Resources, UNITED STATES

## Abstract

**Background/Objectives:**

Sepsis is a severe systemic response to infection with a high mortality rate. A higher incidence has been reported for older people, in persons with a compromised immune system including cancer patients, and in ethnic minorities. We analyzed sepsis mortality and its predictors by ethnicity in the Multiethnic Cohort (MEC).

**Subjects/Methods:**

Among 191,561 white, African American, Native Hawaiian, Japanese American, and Latino cohort members, 49,347 deaths due to all causes and 345 deaths due to sepsis were recorded during follow-up from 1993–96 until 2010. Cox proportional hazard ratios (HRs) and 95% confidence intervals (CIs) were calculated and adjusted for relevant confounders. In addition, national death rates were analyzed to compare mortality by state.

**Results:**

Age-adjusted rates of sepsis death were 5-times higher for Hawaii than Los Angeles (14.4 vs. 2.7 per 100,000). By ethnicity, Native Hawaiians had the highest rate in Hawaii (29.0 per 100,000) and African Americans in Los Angeles (5.2 per 100,000). In fully adjusted models, place of residence was the most important predictor of sepsis mortality (HR = 7.18; 95%CI: 4.37–11.81 Hawaii vs. Los Angeles). African Americans showed the highest risk (HR = 2.08; 95% CI: 1.16–3.75) followed by Native Hawaiians (HR = 1.88; 95% CI: 1.34–2.65) as compared to whites. Among cohort members with cancer (N = 49,794), the 2-fold higher sepsis mortality remained significant in Native Hawaiians only. The geographic and ethnic differences in the MEC agreed with results for national death data.

**Conclusions:**

The finding that African Americans and Native Hawaiians experience a higher mortality risk due to sepsis than other ethnic groups suggest ethnicity-related biological factors in the predisposition of cancer patients and other immune-compromising conditions to develop sepsis, but regional differences in health care access and death coding may also be important.

## Introduction

The incidence of sepsis is, in part, dependent upon age, sex and racial background. In the United States, African Americans and Native Americans present with higher sepsis death rates than whites [1,2]. The Indigenous population of Australia experiences a higher incidence of sepsis compared to the non-indigenous population, e.g., an Australian study of hospital records demonstrated that out of 1,090 patients admitted for sepsis, 50.7% were Indigenous people [3,4]. While there have been no studies on Native Hawaiians and sepsis, this population is at higher risk for death due to cancer and diabetes than other ethnic groups [5]. Cancer is considered an independent predictor of death among sepsis patients as described in a previous report [6]. Immunosuppression often occurs in patients with cancer, which increases the risk of sepsis (16.4 cases/1,000 cancer patients [7,8]). One study using the 1979–2001 National Hospital Discharge Survey found that the occurrence of any type of cancer significantly increased sepsis risk by 10% [9]. Patients diagnosed with brain or lung cancers have an increased risk of developing sepsis [7] and for those with hematological malignancies, the risk is 8.7 times higher than for patients with solid tumors [6]. Overall, the average mortality rate among cancer patients with sepsis is 37.8% representing 8.5% of all cancer deaths that occur annually in the US [7].

Native Hawaiian admixture increases diabetes risk in individuals with mixed ancestry as determined by analysis of the Multiethnic Cohort (MEC). The MEC is a population-based prospective cohort study (*n* = 215,251), which includes African Americans, Latinos, Native Hawaiians, Japanese Americans and whites from California and Hawaii [10]. A previous analysis in the MEC has also demonstrated that obese breast cancer patients of Native Hawaiian ancestry correlates with poorer prognosis compared to other ethnic groups, which may be due to altered expression of key signaling molecules [11]. Indeed, inherited risk factors play a role in infection rates and may, in part, contribute to the incidence and mortality of sepsis [12,13]. In the current analysis, we examined deaths due to sepsis across ethnic groups and explored predictors for sepsis mortality within the MEC to assess whether the risk of dying from sepsis differs by ethnicity and, in particular, if Native Hawaiians experience higher mortality due to sepsis.

## Materials and methods

### Study population and outcome ascertainment

The MEC recruited more than 215,000 men and women, aged 45–75 years at recruitment, in Hawaii and Los Angeles, California during 1993–1996 to investigate dietary and genetic factors for cancer incidence [10]. Participants entered the cohort by returning a 26-page, self-administered questionnaire that asked about medical history, anthropometric measures, diet and other lifestyle factors. A short follow-up questionnaire collected updated information on chronic conditions (heart disease, stroke, diabetes, and hypertension) approximately 5 years later. Although the participants marked all applicable ethnic backgrounds, the following summary categories were created: White, African American, Native Hawaiian, Japanese American, Latino (US born), and Latino (foreign born). The Institutional Review Boards at the University of Hawaii and University of Southern California approved the study protocol.

Mortality information for the cohort members was obtained through state death certificate files and the U.S. National Death Index through December 2010 [14]. Cancer diagnoses were self-reported at cohort entry and identified through linkage to the state-wide population-based cancer registries of Hawaii and California thereafter until December 2010. Specific to the goals of this study, deaths primarily due to sepsis were identified using the International Classification of Disease codes (ICD-9: 038 and 995; ICD-10: A40 and A41). While a previous report [15] using hospital discharge data included all codes for infection and organ dysfunction for sepsis, our goal was to focus only on septicemia.

### Statistical analysis

MEC participants categorized as “Other” ethnic background and those with missing/unknown information for essential variables were excluded from this study, leaving 191,561 of the total 215,000 cohort members for analysis (Table 1). Incidence rates for sepsis death, adjusted to the U.S. 2000 standard population and truncated to ages 45–94 years, were computed by ethnicity. Cox proportional hazards regression with age as a time metric using PROC PHREG in the SAS software package 9.3 (Cary, NC) was applied to estimate hazard ratios (HR) and 95% confidence intervals (CI) for the risk of dying due to sepsis. Observation started at age at cohort entry and ended at death date or the censoring date of 12/31/2010; deaths due to other causes were censored. Independent variables included ethnicity and potential confounders, i.e., age, sex, body mass index (BMI), educational status, the presence of cancer, alcohol intake, smoking history (never, ever, current, and pack years), the presence of chronic conditions (hypertension, diabetes, stroke, heart disease) at cohort entry or follow-up, and geographical region of residence (Hawaii vs. Los Angeles). The proportional hazard assumption was verified using scaled Schoenfeld residuals [16], which demonstrated that the assumption holds for all variables. A second model using the same adjustments was performed for participants who were ever diagnosed with any type of cancer.

**Table 1:** Number of deaths and risk of sepsis death within the Multiethnic Cohort.

	White	African American	Native Hawaiian	Japanese American	Latino	All
Number of participants	48,265	31,919	13,963	55,251	42,163	191,561
Number of deaths	11,691	11,730	3,788	12,332	9,806	49,347
Any cancer diagnosis	12,556	9,639	3,649	14,411	9,539	49,794
Deaths due to sepsis	87	34	66	137	21	345
Hawaii	80	0	66	134	5	285
Los Angeles	7	34	0	3	16	60
Sepsis death with cancer	29	12	21	52	6	120
Age-adjusted death rate^	8.7	5.1	28.6	10.0	1.8	8.3
Hawaii	11.9	--	29.0	12.7	18.0	14.4
Los Angeles	2.1	5.2	--	0.7	1.3	2.7

^Age-adjusted (per 100,000) to the U.S. 2000 standard population and truncated to ages 45–94 years

To compare the results for the MEC with national rates of sepsis mortality, we examined 1999–2014 data for all states using data from the Centers for Disease Control and Prevention. National rates for mortality can be accessed from CDC [17]. We selected deaths with underlying causes of septicemia (ICD-10 codes A40-41) and malignant neoplasms (C00-C96) and applied general linear models to the age-adjusted mortality rates.

## Results

### In Hawaii, Native Hawaiians and, in Los Angeles, African Americans experienced the highest age-adjusted mortality rate from sepsis in the MEC

Among the 191,561 MEC cohort members in the current analysis (Table 1), 49,347 deaths due to all causes were recorded with 345 deaths due to sepsis (285 in Hawaii and 60 in Los Angeles). Age-adjusted rates for death due to sepsis indicated a 5-fold difference between Hawaii and Los Angeles (14.4 vs. 2.7 per 100,000). Native Hawaiians showed the highest age-adjusted sepsis death rates when compared to whites (28.6 vs. 8.7 per 100,000). For Los Angeles, African Americans had the highest rate with 5.2 per 100,000 while the death rates for the other two groups did not differ from whites but were higher in Hawaii than Los Angeles.

After adjustment for other predictors of mortality (Table 2), African Americans had the highest risk of sepsis death (HR = 2.08; 95% CI: 1.16–3.75), which is in keeping with previously reported findings from other assessments within the United States [13,15]. In the adjusted model, Native Hawaiians had the second highest risk of sepsis death (HR = 1.88; 95% CI: 1.34–2.65) while the HRs for Japanese Americans and Latinos did not differ significantly from whites. A cancer diagnosis, obesity, current smoking, and a history of diabetes, stroke, or heart disease were also associated with an elevated risk of dying from sepsis, whereas female sex, higher educational status (>12 years), and light (>0 and <1 drink/day) or heavy (>1 drink/day) consumption of alcohol were inversely associated with sepsis mortality risk.

**Table 2:** Risk factors for sepsis mortality for all MEC participants and cancer patients only*.

		All Participants		Cancer Patients	
Number of participants		191,561		49,794	
Number of deaths due to sepsis		345		120	
Characteristic		HR	95% CI	HR	95% CI
Any cancer diagnosis		1.30	1.04–1.63	-	-
Age at cohort entry (years)		1.01	0.98–1.03	0.94	0.90–0.98
Area	Los Angeles	1.00	-	1.00	-
	Hawaii	7.18	4.37–11.81	7.07	3.01–16.62
Sex	Male	1.00	-	1.00	-
	Female	0.63	0.49–0.79	0.89	0.59–1.34
Ethnicity	White	1.00	-	1.00	-
	African American	2.08	1.16–3.75	2.40	0.88–6.58
	Native Hawaiian	1.88	1.34–2.65	1.92	1.05–3.52
	Japanese	0.89	0.67–1.19	0.95	0.58–1.56
	Latino	0.98	0.53–1.82	1.07	0.35–3.24
Chronic condition	Diabetes	2.12	1.65–2.71	1.95	1.26–3.03
	Heart disease	1.61	1.22–2.12	1.92	1.20–3.07
	Stroke	1.78	1.26–2.51	1.94	1.07–3.53
	Hypertension	0.93	0.74–1.17	1.03	0.70–1.52
Body mass index (kg/m^2^)	<22	1.23	0.88–1.71	1.59	0.97–2.61
	22–<25	1.00	-	1.00	-
	25–<30	0.92	0.69–1.22	0.76	0.47–1.21
	≥30	1.47	1.05–2.05	0.65	0.34–1.23
Smoking history	Never	1.00	-	1.00	-
	Past	1.07	0.79–1.44	0.84	0.50–1.43
	Current	1.81	1.24–2.64	1.59	0.84–3.01
	Pack years	1.01	1.00–1.02	1.01	1.00–1.02
Education (years)	<12	1.00	-	1.00	-
	≥12	0.76	0.60–0.95	0.68	0.46–1.00
Alcohol intake (drinks/day)	0	1.00	-	1.00	-
	0 -<1	0.61	0.46–0.83	0.72	0.43–1.21
	≥1	0.71	0.52–0.97	0.91	0.55–1.48

*Hazard ratios (HR) and 95% confidence intervals obtained from Cox regression using age as time metric and adjusted for all covariates in the table

### Native Hawaiians and African Americans had the highest rate of death due to sepsis among MEC participants diagnosed with cancer or diabetes

Cancer patients are highly susceptible to sepsis due to their suppressed immune system from both the cancer and chemotherapy [8]. As to a cancer diagnosis, 49,794 cohort members were diagnosed with any type of cancer; either before cohort entry or during follow-up; for 120 persons with cancer, the primary cause of death was sepsis (Table 1). In a model restricted to participants ever diagnosed with cancer (Table 2), the elevated risk of dying from sepsis in Native Hawaiians remained significant (HR = 1.92; 95% CI: 1.05–3.52), whereas the 2-fold higher risk for African Americans was not significant any more (HR = 2.40; 0.88–6.58). In addition, three of the chronic conditions and more pack years predicted a higher sepsis mortality, while age at cohort entry and education were inversely associated with sepsis mortality.

Restricting the analysis to only participants who reported diabetes at cohort entry or follow-up (N = 29,917) resulted in similar findings. Area remained the most significant predictor of death due to sepsis (HR = 6.11; 1.16–4.02), Native Hawaiians had the highest risk (HR = 2.16; 0.88–6.58) followed by African Americans (HR = 1.85; 0.64–5.35).

### Sepsis mortality was higher in Hawaii than in California and other States

As the most important predictors in the adjusted models, geographical area (Hawaii vs. California) was highly significant (HR = 7.18; 95 CI: 4.37–11.81; Table 2). Geographical Region (Hawaii vs. Los Angeles, CA) remained the strongest predictor among the MEC participants diagnosed with cancer (HR = 7.07; 95 CI: 3.01–16.62).

The analysis of 1999–2014 national mortality data indicate an overall age-adjusted sepsis death rate of 11 per 100,000 and statistically significant differences across states (p = 0.02) and ethnic group (p<0.001). The respective age-adjusted rates for whites, African Americans, Asian/Pacific Islanders, and Native Americans/Alaska Natives were 10.0, 19.6, 7.3, and 13.2 per 100,000. Separate information on Native Hawaiians is not available in national death data. The rates for California and Hawaii were 3.3 and 13.2 per 100,000, respectively. Using a dataset with multiple causes, i.e., sepsis and cancer, the overall sepsis mortality rate was 5.7 per 100,000 with 7.3 for California and 5.5 for Hawaii for deaths with septicemia and malignant neoplasms as reported causes.

## Discussion

The current findings are based on a large cohort that followed a population with diverse ethnic backgrounds for close to 20 years. Age-adjusted mortality rates due to sepsis were higher for Native Hawaiians than any other ethnic group. However, adjusting for potential confounders, in particular, area of residence resulted in significantly higher risk estimates for African Americans and Native Hawaiians. These findings indicate that these two ethnic groups experienced a 2-fold higher mortality due to sepsis compared to other ethnic groups within their state and suggest that ethnic-related factors may contribute to the susceptibility of developing severe sepsis. Among MEC participants ever diagnosed with cancer or diabetes, only Native Hawaiians showed significantly higher sepsis mortality rates than whites although the risk for African Americans also remained high without reaching statistical significance. Interestingly, sepsis mortality was higher for the State of Hawaii than California within the MEC and according to national data. Similar ethnic differences as in the MEC were present in national death data with the highest rates in African Americans and the lowest in whites.

These findings agree with reports from Australia showing higher mortality from sepsis in indigenous populations [3,4], with previous analyses of national death data showing higher death rates in African Americans [2] and Native Americans [4], and with reports of a 2-fold higher infectious disease death rate in American Indian/Alaska Natives than in whites [18]. The large differences in age-adjusted death rates due to sepsis reported in national death statistics may be due to cancer, obesity, sex, ethnicity, and access to health care [9]. Substantial variations across states, i.e., 41–89 per 100,000, have been described previously, but Hawaii and California rates (58 and 56/100,000) were similar in that analysis [2]. The higher mortality rates as compared to the results presented above are due to a different definition of sepsis based on all infectious diseases [1,2].

This study had several limitations, foremost the fact that only the underlying cause of death and not the contributing causes were available for MEC participants. Therefore, we were not able to assess whether a cancer diagnosis contributed to mortality. At the same time, deaths from cancer with a contributing cause of septicemia could not be identified in the MEC data. Moreover, the total number of sepsis deaths in our dataset was small, limiting the power of our analysis. Also, information about the time of diagnosis and the type of cancer was not taken into account for this analysis. From the literature, it is known that hematologic cancers and cancers of the brain and lung are associated with a high risk of developing sepsis [7]. A future analysis that separates cancer data into types may yield a more accurate representation of the risk of cancer patients dying from sepsis. Further analysis that defines severe sepsis as infection and organ dysfunction, as used in a cohort study on outcomes of severe sepsis [15], may identify more MEC participants that experience death as the result of cancer-associated sepsis, thereby increasing the number of events.

The differences in sepsis mortality across states are difficult to explain; inconsistencies in the health care system and death certification [1,18] may influence how patients were diagnosed in the states of Hawaii and California. Although we restricted our analysis to deaths coded for sepsis (A40-41), previous reports included all deaths due to infectious diseases and yielded much higher mortality rates [1,2]. Investigations that utilized hospital discharge data, often from tertiary hospitals, also used different case definitions [15]. As discussed recently [19], the lack of a clear definition for sepsis and septicemia challenges studies of risk factors and outcomes. This issue is important in determining sepsis incidence and mortality. Changes to the definition of sepsis and updating coding practices have been proposed [20–22]. Unfortunately, outside the national data death, no information on the frequency of bacterial infections in hospitals in Hawaii and California is available.

The findings from the current analysis indicate certain predispositions of cancer patients for sepsis with minority backgrounds and highlight the importance of paying special attention to these patients. A higher infection rate in non-whites and a higher likelihood to develop organ dysfunction has been documented [13], but differences in clinical care as shown for African Americans may also be responsible for the higher sepsis mortality [23]. Identification of ethnicity-related genetic factors to the incidence and prognosis of sepsis may aid in developing more complete sepsis prevention, rapid early detection, and effective treatment approaches.
